# Liquid Metal Enabled Electrobiology: A New Frontier to Tackle Disease Challenges

**DOI:** 10.3390/mi9070360

**Published:** 2018-07-21

**Authors:** Xuelin Wang, Yi Ren, Jing Liu

**Affiliations:** 1 Department of Biomedical Engineering, School of Medicine, Tsinghua University, Beijing 100084, China; wang-xl15@mails.tsinghua.edu.cn (X.W.); reny14@mails.tsinghua.edu.cn (Y.R.); 2 Beijing Key Lab of CryoBiomedical Engineering and Key Lab of Cryogenics, Technical Institute of Physics and Chemistry, Chinese Academy of Sciences, Beijing 100190, China

**Keywords:** electrobiology, liquid metal, soft and conformable electronics, ETFields, disease therapy, health care

## Abstract

In this article, a new conceptual biomedical engineering strategy to tackle modern disease challenges, called liquid metal (LM) enabled electrobiology, is proposed. This generalized and simple method is based on the physiological fact that specially administrated electricity induces a series of subsequent desired biological effects, either shortly, transitionally, or permanently. Due to high compliance within biological tissues, LM would help mold a pervasive method for treating physiological or psychological diseases. As highly conductive and non-toxic multifunctional flexible materials, such LMs can generate any requested electric treating fields (ETFields), which can adapt to various sites inside the human body. The basic mechanisms of electrobiology in delivering electricity to the target tissues and then inducing expected outputs for disease treatment are interpreted. The methods for realizing soft and conformable electronics based on LM are illustrated. Furthermore, a group of typical disease challenges are observed to illustrate the basic strategies for performing LM electrobiology therapy, which include but are not limited to: tissue electronics, brain disorder, immunotherapy, neural functional recovery, muscle stimulation, skin rejuvenation, cosmetology and dieting, artificial organs, cardiac pacing, cancer therapy, etc. Some practical issues regarding electrobiology for future disease therapy are discussed. Perspectives in this direction for incubating a simple biomedical tool for health care are pointed out.

## 1. Introduction

In the cutting-edge field of disease therapy, physiotherapy has received great attention via physical agents such as electricity, magnetism, sound, light, heat, freezing, water, mechanical force, etc. Typical physiotherapy methods generally include: electrotherapy, magnetotherapy, ultrasound therapy, phototherapy, hyperthermia, and cryotherapy [1,2,3]. Compared to traditional disease treatments such as chemotherapy, radiotherapy, and surgical resection, physiotherapy can prevent and treat diseases and restore or reconstruct body function with positive efficacy, minimal invasion, and high survival rate. In particular, electrotherapy has displayed excellent advantages in disease therapy, in some cases preventing or treating cancer [4] via regulating cell apoptosis or proliferation [5].

Electrotherapy, using electrical energy as a medical tool, has been applied for disease treatment since 1855 and has offered significant therapeutic effects [6]. In principle, electrobiology refers to a series of numerous physiological effects caused by electrical stimulation, since the human body is composed of plenty of water and conductive electrolytes. This builds the fundamental of electrotherapy. For example, the activation and inhabitation of cells, such as muscles and cardiac cells, can be controlled by resting potential and action potential [7,8]. Electrical signal is also an important part of neuron communication [9]. Furthermore, electricity can affect the growth and differentiation of cells, and the voltage-gated ion channel is important in substance transportation [10]. Electrical signal can even be found during the photosynthetic process of chloroplasty [11]. Since electrical signal plays such an important role in physiology including psychological events, external electrostimulation (ES) can be applied to influence nearly all the states of cells and tissues and restore some missing functions of injured organs. There are various applications of electrotherapy, including pain management, neuromuscular dysfunction, joint mobility, tissue repair, and acute or chronic edema [6]. However, the development of electrotherapy presents difficulties too. To guarantee the best physiological effects, it is essential that appropriate energy is absorbed by the tissue in question. However, common rigid electrodes generally show less flexibility and biocompatibility, which limits the applications of electrotherapy. Thus, a customizable ES system that depends on different parts of body is necessary. Overall, a novel flexible material—room temperature liquid metal (RTLM), which can match the body disease models perfectly—presents more advantages in electrotherapy, especially for the targets with abnormal shape such as tumors, blood vessels, bones, and cavities etc. [12].

Generally speaking, RTLM refers to liquid metal (LM) whose melting point is around room temperature, such as gallium, bismuth, lead, tin, cadmium, indium, and its alloys [13]. Gallium and its alloys are often applied with different compositions [14]. For example, GaIn_24.5_, the most common LM, refers to the mixture of 75.5% gallium and 24.5% indium by weight. However, mercury, a kind of traditional RTLM, is excluded from ES material because of its high toxicity to the human body. To take advantage of its native properties, RTLM has shown great potential in numerous newly emerging fields, such as microfluidics [15,16], soft and stretchable electronics [13,17], soft robotics [18], biomedical applications [19,20] and the LM catalysis system [21] etc. For instance, Khoshmanesh et al have clearly showcased the desirable attributes of LM in microfluidics, including its interfacial and rheological properties, simple fabrication, flexibility, and various applications (microfluidic components: pumps, valves, heaters, and electrodes) [16]. Liu’s and Dickey’s groups have been working extensively on LM-enabled flexible electronics, and [13,17] highlight its properties (softness, stretchability and electro-conductivity etc.), printing methods (direct writing, mask-based, spray-printing etc.) and applications on liquid metal sensors, e-skin and wearable electronics, and conformable bioelectrodes.

RTLM offers the unique qualities of metallicity and fluidity to allow the manufacture of flexible electronics [14,22], which makes it especially suitable for different electric field shapes within soft human tissues. Compared to non-matal, such as conductive carbon and polymer PEDOT with electrical conductivity of 1.8 × 10^3^ S/m and 8.25 × 10^3^ S/m respectively [23], LM GaIn_24.5_ alloy provides higher electrical conductivity of 3.4 × 10^6^ S/m. In addition, when bending and twisting LM printed wires, the resistance variation is not obvious, which is manifested in its reliability in soft printed electronics [24,25,26,27]. Furthermore, LM has excellent wettability on different substrate materials with different surface roughness and material properties [28]. More importantly, due to the merits of negligible toxicity and benign biocompatibility, such metals have made remarkable progress in bone cement replacement [29], high-resolution angiography contrast [30], gallium-containing anticancer compounds [31], drug delivery nanomedicine [32] and other liquid metal nanoparticles designed for biomedical applications [33,34] etc. Overall, remarkable features of RTLM, such as high electrical conductivity, excellent flexibility, good wettability, and fine biocompatibility, suggest potential for its wide usage in electrobiology for disease therapy [19,20].

In this article, we present a comprehensive interpretation of new conceptual LM electrobiology. A series of potential methods of disease therapy based on biological effects induced by electricity will be outlined. To clarify the foundations of LM that enable electrobiology on human cell, several basic mechanisms are discussed. Finally, we provide an overview of the practical issues of LM-enabled electrobiology, and highlight prospects for further research.

## 2. Foundation of LM-Enabled Electrobiology

### 2.1. Flexibility and Conformability of LM Bioelectrode

**Figure 1:**
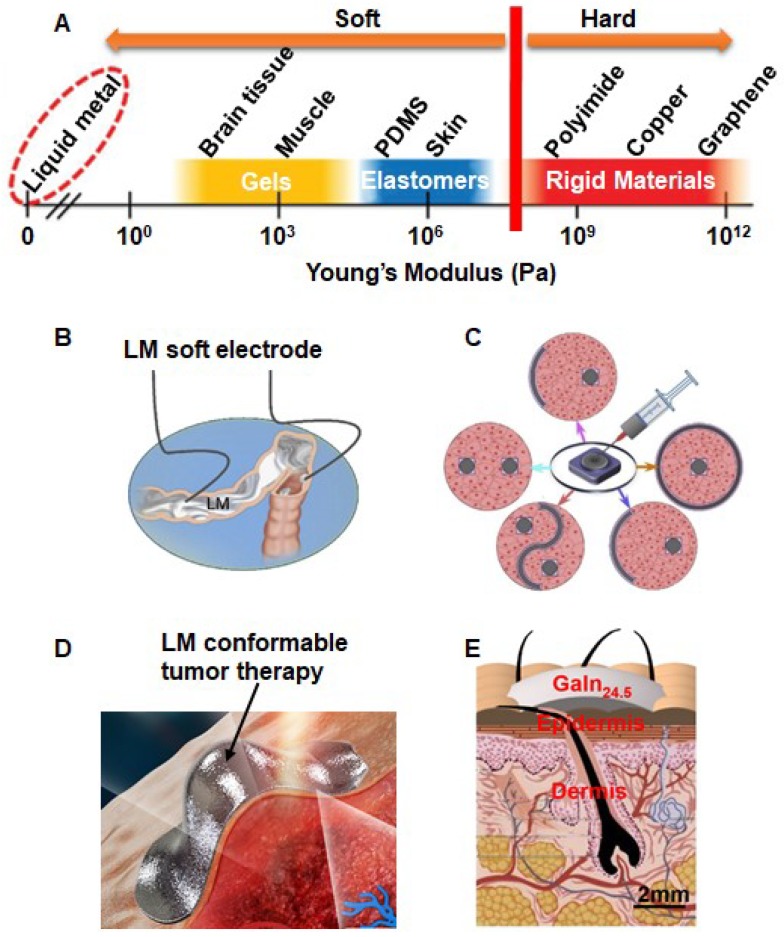
Various flexible and conformable LM bioelectrodes. (**A**) The comparison of Young’s modulus among liquid metal and common soft and rigid materials, reproduced with permission from [17]. (**B**) The conceptual illustration of LM soft electrode, reproduced with permission from [12]. (**C**) LM conformable electrode with five different shapes. (**D**) LM conformable tumor therapy, reproduced with permission from [35]. (**E**) Structural diagram of LM soft electrode on skin surface, reproduced with permission from [36].

From Figure 1A, one can observe that LM has a lower Young’s modulus value than that of rigid materials (polyimide, copper, graphene etc.), elastomers (PDMS (polydimethylsiloxane), skin etc.) and even gels (brain tissue, muscle etc.) [17]. The most flexible material can adapt to complex tissue shapes to achieve any desired biological performance and thus be prepared for various conformable bioelectrodes. Figure 1B depicts a conceptual illustration of a LM soft electrode on a body cavity. Clearly, the flexibility of LM avoids damaging human tissue when used as an electrobiology bioelectrode inside the human body [12]. Meanwhile, LM can be shaped into arbitrary electrode shapes through certain molds (Figure 1C) [12]. Due to its conformability to any complex surface, the LM soft electrode has been introduced to tumor therapy (Figure 1D) [35]. Furthermore, LM can be conformably attached on the skin surface without causing any gap which would significantly reduce the contact resistance between skin and electrode (Figure 1E) and thus provide better signal stability and reduction of noise than conventional Ag/AgCl electrodes [36]. The presentation of numerous LM bioelectrodes suggests that LM owns outstanding reliable flexibility and conformability when employed in LM-enabled electrobiology. However, the biofouling of LM in biological environments is an issue that urgently needs to be addressed. From [37], the LM fed into the digestion system moved as time went on, and finally was eliminated from the body, which showed that LM moves around similar to bodily fluids moved and was difficult to fix.

### 2.2. Electrobiology in Cell Living/Dying

Research has found that electricity can induce cell apoptosis or proliferation, depending on the voltage amplitude and frequency. For instance, electrostimulation greater than 5 V/mm can change the permeability of cell membranes, which increases the rate of cell apoptosis [38,39,40,41,42], while electrostimulation less than 5 V/mm can promote cell proliferation mainly by upregulating growth factors (GF), activating proliferation signaling pathways, increasing intracellular Ca^2+^ and interfering with the cell cycle [43,44,45,46,47,48]. Furthermore, through applying direct current electric fields, cell migration can be induced, which is important in tissue formation, organ regeneration and wound healing [49,50,51,52]. Different factors and signal pathways are involved during these changes. A more detailed description of these mechanisms is displayed in Table 1.

**Figure 2:**
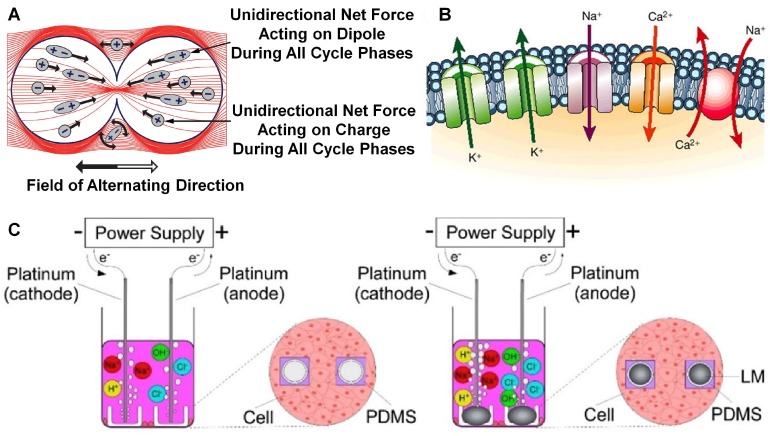
(**A**) The effect of electrical field treatment on dividing cells reproduced with permission from [54]. (**B**) The ion channels of K^+^, Na^+^, Ca^2+^ and electrogenic transporters in cardiac cells reproduced with permission from [57]. (**C**) LM electrode on tumor cell therapy in vitro under electricity reproduced with permission from [12].

Exposing normal or cancer cells to alternating electric fields (1–3 V/cm, 100–300 kHz), dividing cells can be influenced while quiescent cells cannot (Table 1) [53,54]. All charges and polar molecules are forced to alternate direction, which can disrupt the separation of chromosomes (Figure 2A). Traction can be calculated by Bahaj and Bailey theory [55], i.e.,

(1) $$F_{DEP}\infty \frac{V^{2}}{L_{e}^{3}}$$

where $F_{DEP}$ is the dielectric force. V is the applied voltage. $L_{e}$ is the distance between electrodes. The formation of internal structure is damaged, which interferes with cell mitosis and ultimately destroys the cell. LM can be utilized to produce conformable ETFields to enhance the therapeutic effect in electrobiology therapy.

All living cells have transmembrane electrical potential difference and the change of electrical potential controls the opening or closing of ion channels. These voltage-gated ion channels can regulate ion movement across cell membrane to reach a balance [56], and Figure 2B is the voltage-gated ion channel in cardiac cells [57]. When the system is at thermodynamic equilibrium, we can get the transmembrane voltage *V* by Boltzmann distribution [58], i.e.,

(2) $$\frac{P_{i}}{P_{j}}=\exp\left(-\frac{U_{i}-U_{j}}{RT}\right)$$

where, $P_{i}$ and $P_{j}$ are the probability of state i or j, respectively. RT is a constant of the distribution. $U_{i}$ and $U_{j}$ are the state i or j energy, respectively. According to Equation (2), we know the relative energy of an ion X on the inside or outside of membrane [38] which reads as

(3) $$\frac{{\left[X\right]}_{o}}{{\left[X\right]}_{i}}=\exp\left(-\frac{U_{o}-U_{i}}{RT}\right)$$

When I=0,

(4) $$V=V_{X}=\frac{RT}{F}\ln\frac{P_{K}{\left[K\right]}_{o}+P_{Na}{\left[Na\right]}_{o}+P_{Cl}{\left[Cl\right]}_{i}}{P_{K}{\left[K\right]}_{i}+P_{Na}{\left[Na\right]}_{i}+P_{Cl}{\left[Cl\right]}_{o}}$$

where F is the Faraday’s constant (96,480 C/mol). ${\left[X\right]}_{i}$ is the concentration of ion X on the inside of membrane. ${\left[X\right]}_{o}$ is the concentration of ion X on the outside of membrane. When applying electricity on a cell, the resting transmembrane potential can be changed according to Equation (4), which induces cell apoptosis or proliferation [43,59,60,61]. For instance, by inhibiting proliferation and inducing apoptosis, low voltage electric pulse kills the human squamous cell carcinoma cell lines [61].

Furthermore, the LM electrode shows better cell destruction effect than conventional platinum electrodes under electricity with electrochemical effect on tumor cell therapy in vitro (Figure 2C) [12]. Hence, there are many applications of electrobiology in disease therapy, such as cancer therapy [38,39], skeletal muscle atrophy [62], nerve defects [63], wound healing [43], etc. The LM-enabled electrobiology therapy on the human body with high targeting and low side effects changes the cell viability and cell cycle, and promotes or inhibits tissue regeneration, thus realizing functional electrobiology disease treatment [64].

## 3. Basic Therapeutic Strategies of LM Electrobiology

**Figure 3:**
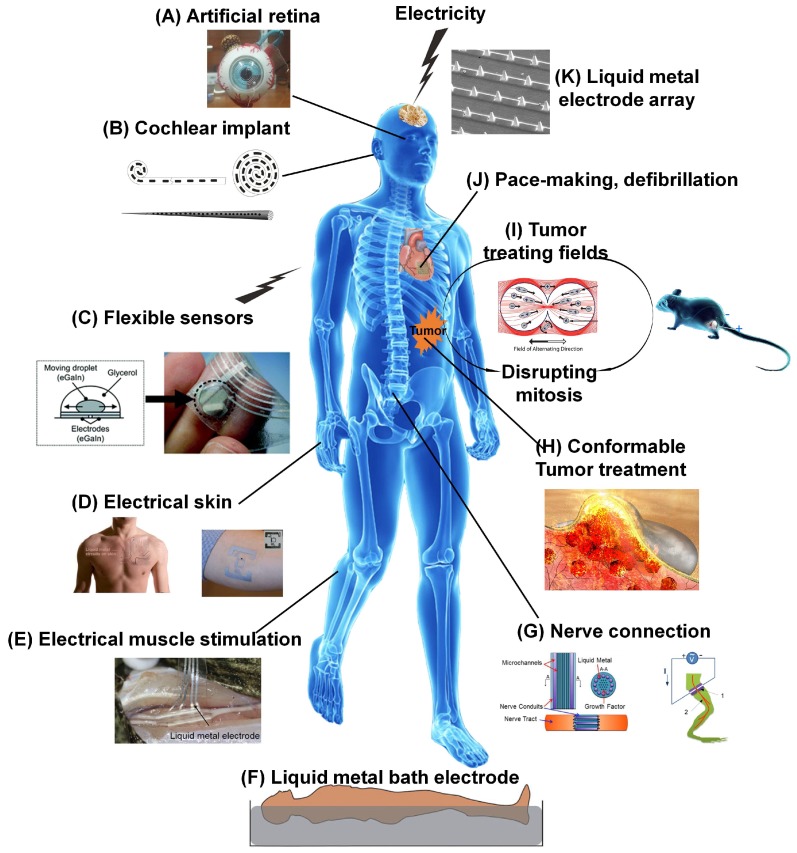
LM-enabled electrobiology in disease therapy. Human model (http://cn.bing.com/); (**A**) Artificial retina reproduced with permission from [69]; (**B**) Cochlear implant [70]; (**C**) Flexible sensors [65]; (**D**) Electrical skin reproduced with permission from [66,73]; (**E**) Electrical muscle stimulation reproduced with permission from [74]; (**F**) Liquid metal bath electrode reproduced with permission from [75]; (**G**) Nerve connection reproduced with permission from [65,76]; (**H**) Conformable tumor treatment; (**I**) Tumor treating fields reproduced with permission from [11,77]; (**J**) Pace-making and defibrillation; (**K**) Liquid metal electrode array reproduced with permission from [78].

The flexibility and electrical conductivity of RTLM give it an important role in electrobiology therapy. In fact, it is gradually becoming used as a bio-material in implantable devices, electrical skin, and wearable bioelectronics for its non-toxicity and benign biocompatibility [65,66,67,68]. As a kind of new flexible electronics, LM has been widely utilized in skin electronics for health monitoring and detection. Beyond skin electronics, LM shows great potential in electrobiology, including brain stimulation, used as a neural junction and for muscle stimulation, cosmetology and dieting, artificial organs, cardiac pacing, cancer therapy, immunization therapy even psychotherapy etc. Figure 3 presents the typical applications and perspectives of LM-enabled electrobiology, such as artificial retinas [69], cochlear implants [70], flexible sensors [71], energy harvesting during movement [72], electrical skin [66,73], electrical muscle stimulation [74], liquid metal bath electrodes [75], nerve connections [65,76], conformable tumor treatment, tumor treating fields [11,77], pace-making and defibrillation, liquid metal electrode array [78], etc. Both experimental studies and clinical trials will enlarge the application scopes of LM-enabled electrobiology therapy.

### 3.1. Brain Stimulation

Electrical brain stimulation (EBS) is designed to stimulate neurons or neural networks in the brain through direct or indirect excitation by electric current, usually for research or therapeutic purposes. If stimulating different parts of the human brain, some acute effects could be induced, including sensory, motor, autonomic, emotional, or cognitive actions [79]. A typical application of EBS is deep brain stimulation, which provides hope to tackle Parkinson’s disease [80], Alzheimer’s disease [81] and major depression and obsessive-compulsive disorder [82]. Furthermore, the current pulse can be applied to damaged nerves to promote nerve regeneration and restore cortical control of functional movement in a human with quadriplegia [83].

**Figure 4:**
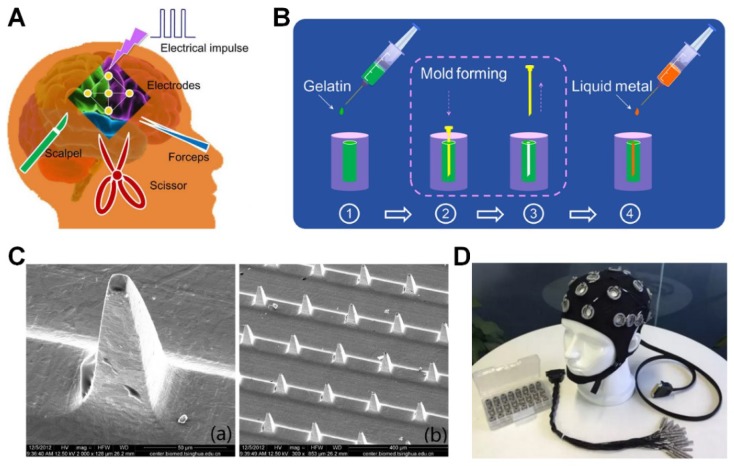
(**A**) Schematics for conventional surgery in implanting electrode for brain stimulator reproduced with permission from [59]; (**B**) The fabrication procedure of injectable metal electrodes reproduced with permission from [65]; (**C**) The SEM image of liquid metal electrode array reproduced with permission from [78]; (**D**) Brain electrode cap (http://cn.bing.com/).

Electrodes for brain stimulation include surface electrodes and implanted electrodes. A wearable brain cap is a typical surface electrode used in electroencephalogram (EEG) recoding (Figure 4D). To achieve more detailed EEG signals and better stimulation effects, implanted electrodes are applied rather than surface electrodes, especially for deep brain stimulation. Conventional implanted bioelectrodes generally require surgery, which often causes severe side effects to patients (Figure 4A) [65]. Therefore, LM is applied to minimally invasive implantable biomedical devices through sequential injections of biocompatible packaging gelation and liquid metal ink (Figure 4B). Because of the solidity of gelatin, various LM-based 3D flexible biological electrodes can be prepared to adapt to different requirements [56]. In addition, Liu et al have manufactured 3D microneedle electrode arrays to acquire electrical signals by LM micromolding technique [78]. The SEM image in Figure 4C indicates its good consistency, which reveals the potential for wider brain electrostimulation application. Due to its miniaturization, the microneedle bioelectrode can effectively decrease the contact resistance with skin, and therefore it can be used to fabricate microtactile sensors to detect weak signal changes. These LM-based implantable 3D bioelectrodes can be utilized in the manufacturing process of novel electrode caps for deep brain stimulation with noninvasive and precise features.

### 3.2. Muscle Stimulation and Neural Functional Recovery

**Figure 5:**
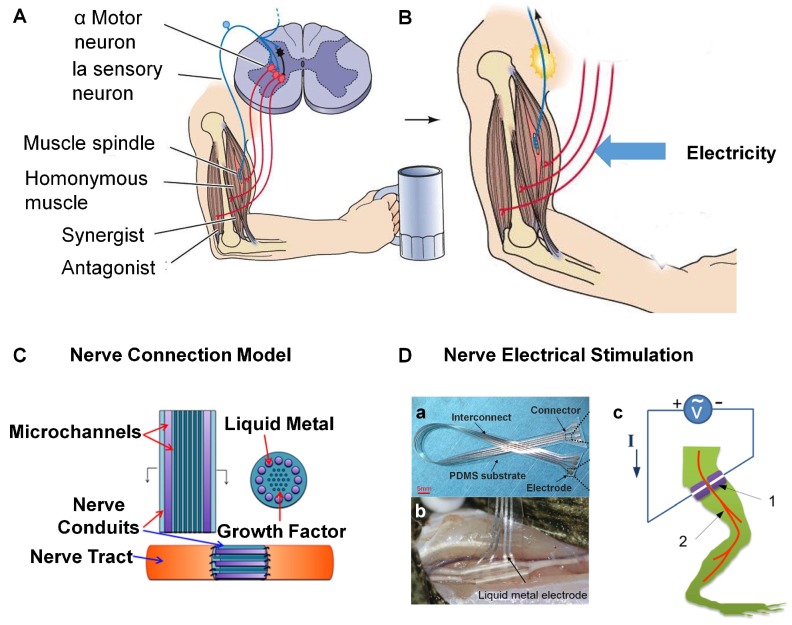
Nerve connection and implanted electrical stimulation. (**A**,**B**) respectively show excitation from central neuron and electricity stimulation to muscle (from NEUROSCIENCE, Fourth Edition, Figure 16.10). (**C**) Nerve connection model with liquid metal microchannels to repair the injured peripheral nerve reproduced with permission from [76]; (**D**) Implanted nerve electrical stimulation based on LM; (**a**) The optical image of LM electrode array reproduced with permission from [74]; (**b**) The implanted LM electrodes for the bullfrog sural nerve and tibial nerve reproduced with permission from [74]; (**c**) Schematic of nerve electrical stimulations based on the injectable LM electrode reproduced with permission from [65].

Electrical muscle stimulation is used as a training, therapeutic or cosmetic tool, including relaxation of muscle spasms, prevention or retardation of disuse atrophy, increasing local blood circulation, muscle re-education, and maintaining or increasing range of motion (Figure 5A,B) [6]. In particular, functional electrical stimulation can be used to generate muscle contraction in patients who have been paralyzed due to injuries of the central nervous system. It has been proven that functional electrical stimulation can help prevent muscle atrophy after major knee ligament surgery [84]. Research on disease prevention through electrostimulation has been conducted and it has been found that electrical stimulation is beneficial for preventing type 2 fiber atrophy in early severe critical illness, and female pelvic floor dysfunction [85,86]. Combining the function of high electric conductivity compared to other soft conductive materials with weak ability of stretching and folding, LM soft electronics designed for operation on the trunk or limbs of the body are attractive for electrical muscle stimulation [66,73,78,87].

**Figure 6:**
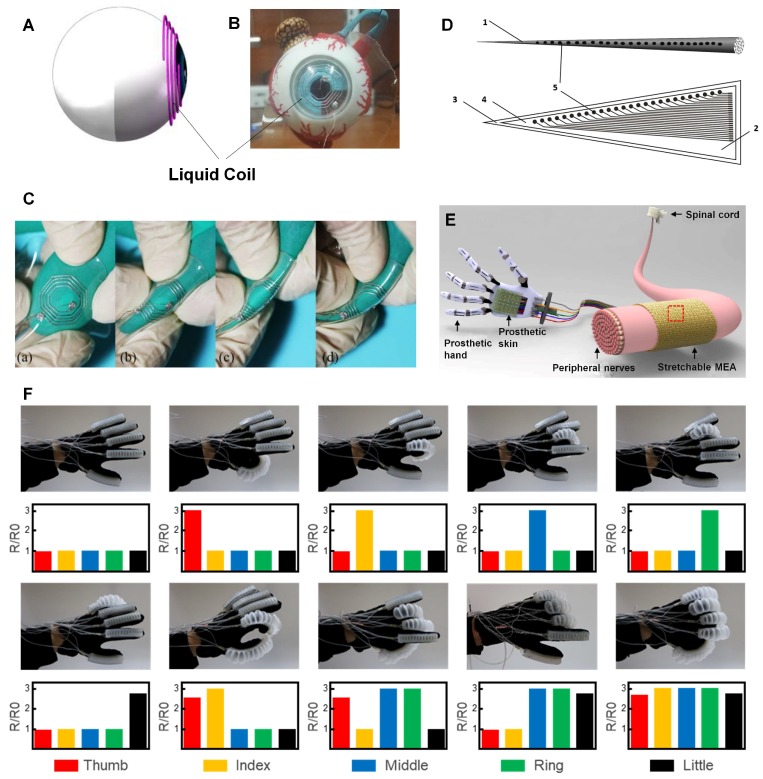
(**A**)The geometric model of LM induction coil. (**B**) The LM coil embedded in PDMS is put on the artificial eyeball for eye movement recording reproduced with permission from [69]. (**C**) Bending the LM coil to show its flexibility reproduced with permission from [69]. (**D**) Cochlear implant electrodes based on LM: (1) A cochlear implant electrode, (2) PDMS membrane, (3) Lower membrane including the microfluidic channels, (4) Upper membrane, (5) Complex of LM and silica gel. (**E**) A prosthetic hand reproduced with permission from [103]. (**F**) The LM-enabled smart flexible pneumatic actuators control the fingers bending at different hand positions reproduced with permission from [104].

Most recently, a kind of LM electrode is developed which is useful in neuromuscular stimulation [74]. As shown in Figure 6D, LM is attached to a flexible PDMS substrate. The implanted LM electrodes are applied to the sural nerve and tibial nerve of a dead bullfrog. The frog leg would go up and down by stimulating sural nerve and tibial nerve alternately, which proves successful neuromuscular stimulation.

Surprisingly, it has also been demonstrated that LM can reconnect broken nerves and restore their function [76], which means neuromuscular electrical stimulation can be applied to not only complete nerves, but also broken ones. As a kind of flexible implant in the neural tissues, LM is applied to connect nerves to achieve long-term performance of implantable neuroprosthesis and neuronal electrical stimulation. Via Galinstan, the present lab recovered the functions of peripheral neurotmesis for the first time, and a novel nerve junction material came into being for patients to regenerate neural networks (Figure 5C) [76]. The authors built up the bullfrog gastrocnemius model and introduced LM to link the snipped neural tissue. With a weak electrical stimulation, they proved the advantage of LM in nerve conduction. The result shows that the neural model connected by LM has a good stimulation signal which maintains the same consistency and fidelity as the normal neural tissue before cut. Due to its strong contrast under X-ray, LM can easily be taken out of the body after completing nerve repair to avoid secondary surgery. This method opens a new direction for neural connections and restoration.

### 3.3. Beauty and Weight Loss

Skin care and weight loss are increasingly popular nowadays. As the visible organ with multiple physiological functions, skin has been a hot topic in youthful study [88]. In addition, obesity is closely related to both beauty and health, thus attracting increasing attention. It has been found that electrical stimulation has promising potential in beauty therapy [89].

Painting LM on skin surface, electrical stimulation induces cell proliferation to make skin a better protective barrier that mitigates exposure to external factors such as extreme temperature, microorganisms, and radiation. Consequently, youthful and healthy skin can appear via electrostimulation treatment. Furthermore, stimulating facial muscles and skin can improve circulation, activate fibroblasts and tone flaccid and sagging facial muscle [89]. Conformable LM can even stimulate facial skin for better elasticity and smoothness. Using the spray-printing method, LM can be directly printed on the skin based on the mask shape. From [90], someone exposed to gallium for a long period may experience dermal inflammation and gallium poisoning. Consequently, we should strictly control the time for which LM is painted on the skin to avoid the gallium poisoning.

Brown adipose tissue (BAT) is a heat-producing organ that plays an important role in decomposing human white adipose tissue (WAT) to reduce the risk of obesity [91,92,93,94]. As well as exercising, dieting or medicine regulating [95], electrical stimulation has been applied to active BAT thermogenesis with microcurrent on the dorsal surface of the tissue [96,97,98,99,100]. Due to the low-fidelity coupling between the traditional rigid metallic electrodes and dorsal skin surface [101,102], RTLM is expected to be painted on the skin to reduce the energy loss on electrostimulation obesity.

It is also believed that electrical stimulation with wearable LM-based electronics makes the users more likely to participate in exercise, since ES devices are comfortable and effective when doing sports. It allows the body to activate muscles without strain or stress and reduces the risk of injuries [89]. Combining LM-based wearable electrostimulation devices with proper exercises, healthy weight loss can contribute towards solving weight problems.

### 3.4. Artificial Retina and Cochlear Implantation

Amit et al. realized a soft coil based on LM and demonstrated that this coil is an excellent alternative to stiff and uncomfortable coils for biomedical implants [69]. LM was injected into elastomeric microfluidic channels to produce LM-based coils, which can deliver power to the implant coil efficiently in a telemetry system. Meanwhile, Zhao et al. designed an innovative LM electronic coil for eye movement tracking (Figure 6A,B) [69]. Experiments show that the results measured by LM coil correlate well with those of classical copper coil during eye movement. Meanwhile, Figure 6C has fully showcased the excellent flexibility of LM coil. Coupled with its flexibility, these coils show great advantages in an implantable system, such as a retinal prosthesis system, whose secondary coil size is strictly limited by the anatomy of eye. Based on these studies, LM-based coils may hopefully be applied to wireless battery chargers, cardiac implants, and glucose-monitoring implants.

Stefan et al. designed a new cochlear implant electrode with LM [70]. Commonly, the cochlear implant system transfers auditory signals to brain by activating auditory nerves. Unlike typical cochlear implant systems, this design applies LM to electrode wires, with higher flexibility and biocompatibility. Furthermore, the array carrier used in this design is reactive to LM, which makes sure that a local leakage seal will form when a break occurs so that LM will not migrate to the outer surface. Another LM-based cochlear implant electrode incorporating microfluidic is designed with good conductivity and flexibility to break the limitation of tradition electrodes (Figure 6D). The electrode is very thin and soft so that it does little physical damage to the internal structure of cochlea. As a result, residual hearing can be protected.

### 3.5. Prostheses

Recent research has been conducted to apply LM to prostheses. Prostheses can be divided into two types: aesthetic functional devices and myoelectric devices. In particular, myoelectric prostheses work by sensing electrical signals from nerves and muscles via electrodes. Combining LM electrodes with signal process systems, the motion of prostheses can be controlled by users precisely. To meet even higher standards, a smart prosthetic hand should not only receive the instructions from the nervous system and make corresponding responses, but also feel various types of external stimuli and transmit signals to the corresponding peripheral nervous system to realize better control (Figure 6E) [103,105]. For this purpose, artificial sensing skin and myoelectric prostheses can be combined to makes the prostheses dexterous and smart.

With the development of microfluidic technologies, LM can be used to produce soft tactile sensors, which has great potential in sensing skin, such as temperature, sweat analysis, skin patches and pressure, based on the change of resistance and capacitance. For instance, Jung et al. combined microfluidic channels and LM together to make a pressure sensor [106]. Additionally, an inertial sensor has been designed based on the capacitance between two LM electrodes, which can measure tilt angles and record arm gestures [71]. Guo et al have developed a smart flexible pneumatic actuator based on LM amalgams [104]. The bending station of the actuator can be controlled by aeration and measured by the LM sensor printed on the elastic materials. Figure 6F shows the electrical resistance change measured by LM bending sensors when the five actuators on the fingers are filled with different amounts of gas. The wavy or serpentine electronics in Rogers’ group are often connected by Cu antenna, a conventional rigid material, and therefore the serpentine shape is fabricated to ensure the flexibility and stretchability of the electronics [107]. When applying LM to prepare soft electronics, we can directly print it as a straight line due to its fluidity and infinite softness. However, it is difficult for LM to produce high-precision devices and device performance needs to be improved. These designs in soft tactile sensors promote advances in artificial-sensing skin and increase the possibility of successfully applying artificial sensing skin to prostheses. It can be concluded that with soft and stretchable LM sensors and electrodes, the future of a comfortable and smart prosthesis for amputees is promising.

### 3.6. Cardiac Pacing

For the treatment of heart-rhythm diseases, advanced capabilities in electrical recording and stimulation are essential [108]. Cardiac electrotherapies include pacing, defibrillation, and cardiac ablation therapy [109]. Stretchable electronics show high performance in cardiac electrotherapies with the development of new materials.

**Figure 7:**
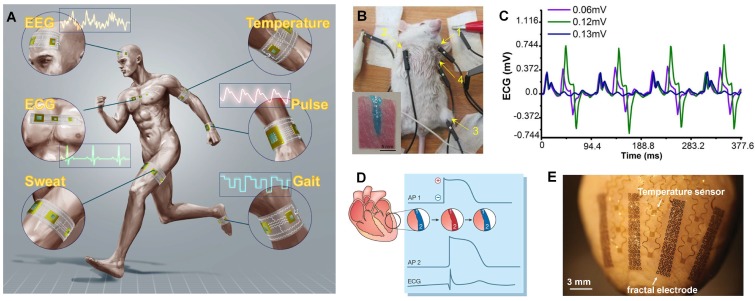
(**A**) The intelligent electronic skin network based on LM e-skin and wearable bioelectronics [110]. (**B**) Illustration for conducting an electrical stimulation to the experimental mouse reproduced with permission from [65]. (**C**) The recorded ECG signals of experimental mouse undergoing a 10 Hz electrical stimulation with magnitude of 0.6 mV, 1.2 mV and 1.3 mV, respectively reproduced with permission from [65]. (**D**) The formation of ECG signals; negatively polarized (resting) muscle is shown as blue; depolarized muscle is red; Action potentials recorded at sites 1 and 2 are similar in timing and morphology reproduced with permission from [57]. (**E**) Image of a representative device integrated on a Langendorff-perfused rabbit heart. The white arrows highlight functional components reproduced with permission from [109].

Combining with specific sensors or chips, multifunctional soft devices are produced. Attaching the device onto soft skin, stable physiological signals (such as EEG (electroencephalogram), ECG (electrocardiogram), sweat, temperature, pulse, and gait etc.) can be detected in human activities. An intelligent electronic skin network (Figure 7A) based on LM flexible electronics is expected in the future with the functions of sensing, monitoring, and treating [110]. Jin et al proposed the original idea of 3D injectable electronics and injected an LM electrode into the left side of the thorax near the left upper limb of a mouse. Then, electrical stimulation with different magnitude (0.6 mV, 1.2 mV and 1.3 mV) was applied to the mouse and the ECG signal was recorded [65]. When magnitude increased, PQ interval of ECG signal shortened and heart rate increased (Figure 7C). The phenomenon that the increase of electrical stimulation induced the spasm of mouse muscle has inspired the applications of implanted electrical stimulation on heart pace-making and defibrillation. ECG signals are initiated by a pulse of electrical excitation from the specialized pacemaker cells, which makes the heart beat rhythmically. The repolarization of the heart cell (Figure 7D site 1) is interrupted by depolarization (Figure 7D site 2), enabling the electrical impulse to spread from cell to cell in the syncytial heart [57]. Additionally, stretchable wireless power transfer devices can be prepared by LM circuits with excellent flexibility and stable energy conversion [111]. All these results provide the possibility that LM can be applied to recode cardiac activity and deliver electrical stimulation to treat cardiac diseases (Figure 7E) [109].

### 3.7. Tumor Treating

**Figure 8:**
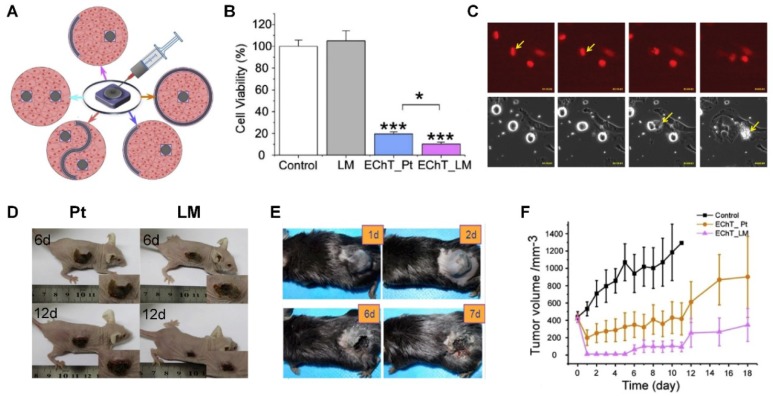
Electrostimulation in tumor treatment. (**A**) Five therapeutic method configurations of LM electrode in different shapes reproduced with permission from [12]; (**B**) Cell viability of LM, EchT_Pt, EchT_LM reproduced with permission from [12]; (**C**) The microscopic evidence of HeLa cells membrane blebbing with TTFields treatment during mitosis reproduced with permission from [114]; (**D**) The 6th and 12th days treatments of melanin tumor-bearing mice by Pt electrode and LM electrode treating reproduced with permission from [12]; (**E**) Electrical stimulation treatment of malignant melanoma tumor on C57BL/6 mice based on LM spray-printing reproduced with permission from [77]; (**F**) Tumor volume of different groups of tumor-bearing mice reproduced with permission from [12].

Some in vitro evidence has demonstrated the efficacy of tumor-treating electrical fields on cancer therapy, such as human melanoma, glioma, lung, prostate, pancreas, and breast; mouse adenocarcinoma and melanoma; and rat glioma [54,112,113]. From Section 2, we can draw the conclusion that electrostimulation in cancer therapy mainly inhibits and kills tumor cells through two mechanisms: disrupting cell mitosis and increasing cell apoptosis. Li et al. sprayed LM on a malignant melanoma tumor in C57BL/6 mice and added a sine wave electrical current to deliver electrical stimulation to tumor tissue [77]. After one week of treatment, the tumors treated by 2 V/cm, 300 kHz and 1.6 V/cm, 300 kHz electrical stimulation diminished (Figure 8E). Unlike Li et al.’s spray-printing method, Sun et al. put forward a new injection method. Through injecting, the shape of LM electrodes is easily changed to achieve better performance of tumor treatment (Figure 8A). Comparing in vitro cell viability (Figure 8B) and in vivo antitumor effects (Figure 8D) of conformable LM electrodes and conventional platinum (Pt) electrodes, LM electrodes provide not only transformable capabilities, but also a highly efficient electron-transfer ability, which allows better tumor destruction effects in both anode and cathode areas (Figure 8F) [12]. It is hopeful that LM electrodes can help to regulate various desirable configurations, in two or even three dimensions, which can be applied to irregular tumor surfaces. Furthermore, the electrical field in tumor treatment can destroy tumor cells with no appreciable heat effect or stimulation on surrounding muscles and nerves significantly. Different from rigid electrodes or sol-gel electrode materials, the LM sprayed on skin provides the biggest surface area, which diminishes the contact resistance and therefore reduces the burning rate of electrical stimulation sites.

In addition, Figure 8C displays the microscopic evidence of HeLa cell membrane blebbing with electrical field treatment during mitosis [114], which reveals violent structural disruption. The promising results offer evidence to electrical stimulation by LM electrodes for further cancer therapy.

### 3.8. Immunotherapy and Psychotherapy with LM Bath Electrode

Immunization therapy in the present paper refers to the enhancing or inhibiting of an organism’s immune system due to the introduction of electrical stimulation to achieve a desired therapeutic output. Unlike conventional immunization therapy through injecting immunostimulant or immunosuppressive drugs into a patient’s body, the novel immunization therapy will enable minimally invasive disease therapy with no drugs to inject into human body and applying an LM bath electrode to conformably and electrically stimulate the organism [75]. Besides, when electricity stimulates the human body, we will have lots of strange feelings. Regarding virtual reality, LM-enabled electrobiology can create a virtual environment and regulate a human’s emotions, and could therefore be used in psychotherapy. Imagining the future life, a patient can enjoy his treatment of whatever physical or psychical diseases in the LM bath with specific amplitude and frequency electricity stimulation.

**Figure 9:**
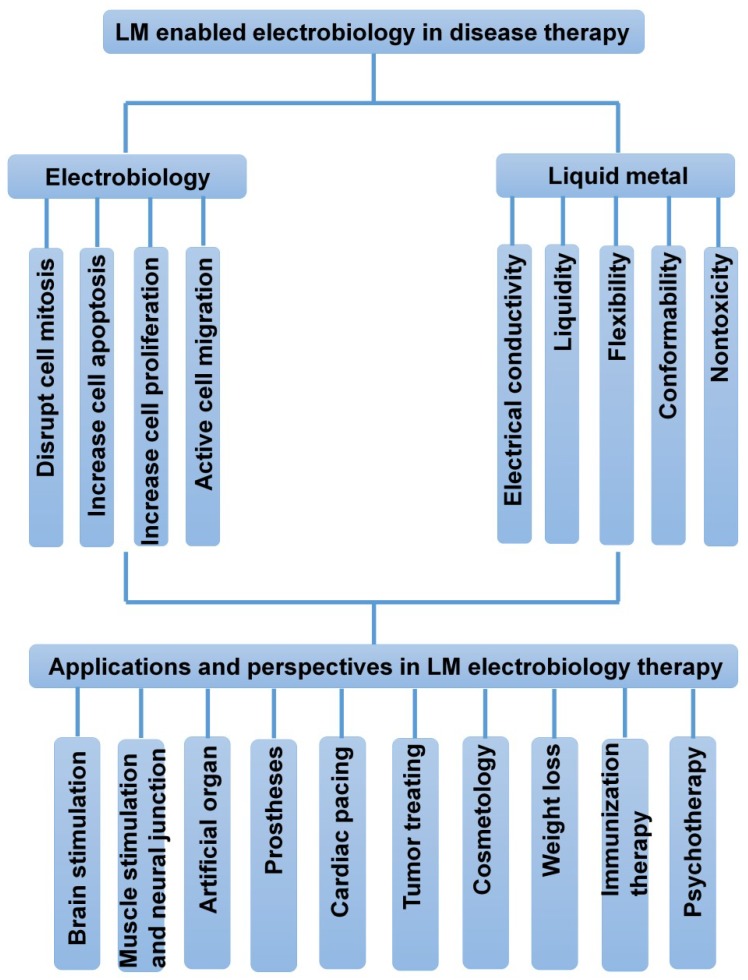
The summary diagram of LM-enabled electrobiology in disease therapy.

Overall, considering all the above potential routes to tackle modern disease challenges, we can summarize the generalized way of administrating LM-enabled electrobiology as Figure 9. There is plenty of space to explore these areas in the future.

## 4. Conclusions

After excluding the well-known poisonous liquid metal mercury, gallium-based LMs are considered as new emerging biomaterials due to their widespread applications in biomedical engineering. However, their metallic conductivity, negligible toxicity, low vapor pressure and high mechanical conformity mean that there are still many urgent problems to be solved [13,17,18,20,115]. Plenty of in vitro and in vivo experiments have demonstrated that LM does not reduce cell viability and causes the inflammatory reaction of mouse skin [12,35,36]. However, a previous report has shown that without external agitation, Ga-In liquid metal in an aqueous environment was rather safe with only Ga being detected. Otherwise, with some mechanical agitation, In ion could be detected with biological toxicity [116]. Ivanoff et al have reported a gallium poisoning case with arms, eyes, and ears of the patient diagnosed with allergic dermatitis, headaches, tachycardia, and difficulty in breathing [90]. Meanwhile, inhaling gallium could cause liver and kidney dystrophy, and lung damage [90]. Therefore, people who work with gallium and its compounds should be careful to avoid gallium poisoning.

The targeted distribution and fixation of LM in living organisms as well as its complete elimination from the body are difficulties. Some researchers have suggested the syringe injection method to alleviate a patient’s suffering [12,32], but residues of LM in the body could pose potential risk for people’s health [37]. In addition, the diverse transformation of LM under the control of an electric field might have some unexpected effect on LM electrobiology therapy [117]. Its oxidization, encapsulation to different shapes, the LM leak from the packaging layer, and cost are all the things we should consider in applications of LM electrobiology treatments [20,115].

In summary, the basic concept of LM electrobiology is proposed and a generalized disease therapy framework is established for coming studies and clinical clarifications. Due to its remarkable material features, such as metallicity, reliable flexibility, excellent electrical conductivity, and non-toxicity, LM can not only adapt to body disease models to generate conformable electrical treatment, but also reduce the contact resistance to avoid more energy loss when performing the task. At this stage, several basic electrostimulation mechanisms in disease therapy have been investigated. One of them cures diseases by inducing cell proliferation, migration, and excitation, including electrical muscle stimulation, nerve connection, artificial organs, cardiac pacing etc. The others prevent the cell lines by disrupting cell mitosis or increasing cell apoptosis. Taking oncotherapy as an example, under a specific tumor-treating field for a period of time, the tumor diminishes along with cancer cells being killed. Overall, given specific design, typical applications of LM electrostimulation would provide satisfactory therapeutic efficacy, a conformable treatment area and biological availability. This may lead to evermore simple physical method for future clinics and health care.

## Figures and Tables

**Table 1:** Effect of electrobiology on cells and related mechanism.

Effect	Electrostimulation	Mechanism	Refs.
Disrupt Cell Mitosis	1–3 V/cm, 100–300 kHz	(1) In mitosis cells, the non-uniform distribution of electric field induces high electric field intensity in the junction of two daughter cells (Figure 3A). All charges and polar molecules are forced to alternate direction, which can disrupt the separation of chromosomes. (2) Because of the uniform distribution of electric field in quiescent cells, there is almost no damage to normal cells.	[53,54]
Increase Cell Apoptosis	Greater Than 5 V/mm	(1) ES makes excessive Ca^2+^ influx due to the pore formation/electroporation in cell membrane. (2) Caspase 3, a key player in apoptosis, is activated. (3) The damaged DNA induces MEK1/2 phosphorylation. Then the activated ERK1/2 by phosphorylated MEK1/2 prevents cell proliferation by sequestering ERK1/2 in cytosol, which can increase cell cycle progression from G to S phase	[5,38,39,40,41,42]
Increase Cell Proliferation	Less Than 5 V/mm	(1) ES increases the secretion of growth factors. (2) Akt prevents cell apoptosis by inhibiting the pro-apoptotic factors Bim, caspase 9, Bax, Bad and FOXO-3. In addition, Akt promotes the cell survival by activating transcription factor NF-KB to advance transcription of pro-survival genes. (3) ERK1/2 is translocated to the nucleus to activate G to S phase transition.	[5,43,44,45,46,47,48]
Activate Cell Migration	Migration Velocity Depends on Cell Type and Voltage Amplitude	(1) NaKA and NHE3, considered as a switch of cathodal/anodal migration, accumulates at the cathodal or anodal edge of the cells, causing cell depolarization and cytoskeleton redistribution. (2) PI3K, which is found to accumulate and activate at the leading edge of cells with downstream effectors, induces cell cathodal migration. (3) PTEN is more likely to activate at the anodal side rather than cathodal side.	[49,50,51,52]
